# Trends in sociodemographic inequalities in prenatal care in Baixada Litorânea, a region of the state of Rio de Janeiro, Brazil, 2000-2020: an ecological study

**DOI:** 10.1590/S2237-96222022000300006

**Published:** 2022-11-04

**Authors:** Sandra Costa Fonseca, Zenair Simião Barbosa de Carvalho, Pauline Lorena Kale, Cynthia Boschi-Pinto, Júlia Cardoso Correia Guimarães

**Affiliations:** 1 Universidade Federal Fluminense, Instituto de Saúde Coletiva, Niterói, RJ, Brazil; 2 Universidade Federal do Rio de Janeiro, Instituto de Estudos em Saúde Coletiva, Rio de Janeiro, RJ, Brazil; 3 Universidade Federal Fluminense, Faculdade de Medicina, Niterói, RJ, Brazil

**Keywords:** Prenatal Care, Healthcare Disparities, Quality of Health Care, Time Series Studies

## Abstract

**Objective:**

To analyze trends in sociodemographic inequalities in the access to and use of prenatal care in Baixada Litorânea, a region of the state of Rio de Janeiro, Brazil, 2000-2020.

**Methods:**

This was an ecological time-series study of the number of visits and adequacy of access to prenatal care. Absolute (differences) and relative (ratios) inequalities were calculated between extreme categories of variables; trends were estimated using joinpoint regression.

**Results:**

A total of 185,242 pregnant women were studied. A proportion of ≥ 7 visits increased annually by 2.4% (95%CI 1.1;3.7) between 2013 (54.4%) and 2020 (63.4%), stable for less than eight years of schooling. Adequacy of access increased 2.6% (95%CI 1.2;4.0) between 2014 and 2020, stable for women ≥ 35 years old and schooling ≥ 12 years. Absolute inequalities decreased (between 3.5% and 6.4%) for age and race/skin color, and relative inequalities decreased (between 7.7% and 20.0%) for all variables.

**Conclusion:**

Access and number of prenatal consultations increased, however, remained lower for adolescents, women with low level of schooling and those of Black and mixed race/skin color.

**Study contributions**

**Main results**

Sociodemographic inequalities in the performance of seven or more prenatal consultations with pregnant women have decreased in Baixada Litorânea, however, the adequacy of access and number of visits remain lower for adolescents, women with low level of schooling and those of Black and mixed race/skin color.

**Implications for services**

Since the Maternal-Child Health Services management is the responsibility of the health region, the dissemination of these results may support efforts to expand the coverage of qualified prenatal care in Baixada Litorânea aimed at the most vulnerable women.

**Perspectives**

Managers and health professionals should direct efforts to reduce inequalities in prenatal care. Monitoring indicators according to sociodemographic variables should continue, and the study may contribute as a basis for future evaluations.

## Introduction

Prenatal care plays a fundamental role in promoting a healthy pregnancy for every woman, fetus and newborn.^1^ ^-^ ^3^ Adequate prenatal care identifies possible risk situations for the mother-child binomial and increases the chances of timely interventions to promote adequate nutrition, prevent vertically transmitted diseases, and control maternal morbidities, such as arterial hypertension, diabetes *mellitus*, and urinary tract infection.^2^ ^,^ ^3^

Prenatal care coverage in Brazil is almost universal. However, the adequacy of care for pregnant women has not been fully achieved yet,^4^ ^,^ ^5^ thus, it needs to be improved in relation to the number of visits, tests and procedures, as recommended by the Maternal-Child Health Services strategy.^5^ This initiative, created in 2011, has expanded humanized care during pregnancy and childbirth, strengthening national programs aimed at women's and children's health.^5^ Regarding the prenatal component, the strategy provides for reception, risk and vulnerability classification, expanded access and improvement of prenatal care quality.

The adequacy of prenatal care has been carefully studied, based on different approaches and definitions of adequate prenatal care.^4^ However, there is no consensus on the concept of adequacy, and simple, quantitative criteria such as the number of prenatal care visits or more complex criteria, including exams and procedures, infrastructure, and a binding between the pregnant woman and a maternity hospital can be used.^4^ ^,^ ^6^ ^-^ ^8^

Studies evaluating the number of consultations as a quality indicator, on the dimension of use of prenatal services,^9^ ^-^ ^12^ may use secondary data, such as those from the Live Birth Information System (*Sistema de Informação Sobre Nacidos Vivos* - SINASC), publicly available and updated. The Ministry of Health proposed an adequacy of access indicator, which takes into account the number of visits and the beginning of prenatal care, also available on SINASC.^13^

In two national evaluations of prenatal care, based on the number of visits and the beginning of follow-up care,^12^ ^,^ ^13^ the Southeast region showed a good performance. The state of Rio de Janeiro reported the lowest average number of prenatal visits and the lowest percentage of women starting prenatal care during the first trimester,^12^ in addition to lower proportions of the index of adequacy of access to health care,^13^ when compared to other states in the region. We could not identify any recent studies, after the implementation of the Maternal-Child Health Services, which had analyzed the number of visits and/or the beginning of prenatal care in different locations in the state, besides the capital. However, the reports of the State Department of Health show a lower proportion of women who has had seven or more prenatal care visits in regions outside the metropolitan area, such as Baixada Litorânea.^14^

In addition to adequacy, it is essential to study inequalities in prenatal care. Studies show that both the number of visits and other measures of adequacy of prenatal care can be socially determined.^15^ ^-^ ^18^ In this context, the identification of populations with limited access to and/or adequacy of care may support strategies for health policies aimed at reducing inequalities, both in specific health programs and in intersectoral ones.^5^ ^,^ ^17^ ^,^ ^19^ Adequate interventions during prenatal care and high coverage of neglected populations effectively reduce maternal and infant morbidity and mortality.^2^ ^,^ ^17^ ^,^ ^19^

Adolescence, low level of schooling and Black and mixed race/skin color are maternal characteristics often related to disparities in maternal and child health care, such as limited access, fewer prenatal care visits and unfavorable perinatal outcomes.^15^ ^-^ ^18^ ^,^ ^20^ ^-^ ^22^ Data on age, schooling and race/skin color provided by SINASC, enable this type study.

The aim of this study was to analyze trends in sociodemographic inequalities in the access to and use of prenatal care in the health region of Baixada Litorânea, state of Rio de Janeiro, Brazil, between 2000 and 2020.

## Methods

An ecological time-series study was conducted for the period 2000-2020, in Baixada Litorânea, a region of the state of Rio de Janeiro. The fraction of time "year"^23^ was considered as the unity of analysis. Each indicator was evaluated annually, and its temporal variability estimated.

Baixada Litorânea fluminense, with 823,899 inhabitants, includes nine municipalities: Araruama, Armação dos Búzios, Arraial do Cabo, Cabo Frio, Casimiro de Abreu, Iguaba Grande, Rio das Ostras, São Pedro da Aldeia and Saquarema.^14^ The human development index (HDI) of the municipalities ranges from 0.709 (Saquarema) to 0.773 (Rio das Ostras). The population coverage provided by Primary Health Care within the Brazilian National Health System (*Sistema Único de Saúde* - SUS) in the region was 67.5% in 2017; Araruama, Rio das Ostras and Saquarema showed the lowest percentages. Baixada Litorânea showed the lowest percentage of having seven or more prenatal consultations in the state of Rio de Janeiro.^14^

The study population was comprised of women living in the region, who delivered live births in single pregnancy, equal to or greater than 22 weeks, weighting more than 500g.

SINASC was the source of data, available in the websites of Rio de Janeiro State Department of Health (SES/RJ) and the Brazilian National Health System Information Technology Department (DATASUS) of the Ministry of Health, accessed in April 2022.^24^ ^,^ ^25^ Live Birth Certificate (LBC), an instrument whose data serve as the basis for SINASC, underwent a change in 2011, including the expansion of sociodemographic, reproductive and health care information (beginning of prenatal care, detailed number of visits, relationship between labor and cesarean section).^26^ LBC fields related to maternal age and schooling were previously disabled, and filled in only by categories; as of 2011, open fields were included, providing more detailed information. The SES/RJ and DATASUS websites provide information on sociodemographic variables, both in detail and by category, enabling comparison between periods before and after 2011.

We analyzed the following variables

- Maternal sociodemographic variable, categorically, according to the availability of the TabNet version of SINASC:
  - Age (in years: up to 19; 20 to 34; ≥ 35).
  - Schooling (in years of study: 0 to 3; 4 to 7; 8 to 11; and 12 and more).
  - Race/skin color (White; Black; mixed race; Yellow; Indigenous).
- Prenatal care, according to two indicators:
  - Use of prenatal care - only the variable "number of prenatal care visits", information categorized and available for the entire period analyzed was taken into consideration; we adopted as a cutoff point, having seven or more prenatal care visits, a number compatible with SINASC categorization and closer to the recommendation of the World Health Organization (WHO).^1^
  - Adequacy of access to prenatal care, defined according to the recommendation of the Ministry of Health, based on information on the number of visits and the period when prenatal care started, such as, (i) "Did not" (no prenatal care visits), (ii) "Inadequate" (started after the 3^rd^ month and/or fewer than three prenatal care visits), (iii) "Intermediate" (started before or during the 3^rd^ month and three to five prenatal care visits), (iv) "Adequate" (started before or during the 3^rd^ month and six prenatal care visits) and (v) "More than adequate" (started before or during the 3^rd^ month and seven or more prenatal care visits);^13^ records with missing or ignored information on the number of visits and the beginning of prenatal care comprised the category "Not classified".^13^

Since 2014, the variable "adequacy of access to prenatal care", available in the national SINASC database (in DATASUS), has been categorized into five groups as described in the previous paragraph. The adequacy of access according to maternal age, schooling and race/skin color was analyzed. Taking into consideration the increasing order of quality of the variable, the proportions of extreme situations were compared: "did not/inadequate" or "adequate/more than adequate", without considering, therefore, the intermediate category.

Absolute distributions and the annual proportion of adequacy of access for the total number of live births and those with gestational age equal to or greater than 37 weeks (full-term) were described. Other adequacy indicators adjust the number of prenatal care visits for gestational age (GA), because it is common knowledge that the lower the GA, the shorter the time to obtain the ideal number of prenatal care visits. As this indicator - adequacy of access - does not include this variable in its formulation, the total number of live births at ≥ 37 weeks gestation was analyzed, enough time to reach seven prenatal care visits. The indicator "adequacy of access" was analyzed only for the period available in the national SINASC database, from 2014 to 2020.

For the statistical analysis of the time series, we used the Joinpoint Regression program, which adjusts, on a logarithmic scale, linear trends and changes in these trends (joinpoints). For the significance test, the Monte Carlo Permutation Method, which adjusts the best line of each segment, was used. When these segments are established, the direction and magnitude of the estimated trends are represented by their respective annual percentage change (APC). The APC is calculated as follows:

APC=100 X (It+1-It)/I

where I is the indicator in the year (It) and in the following year (It +1). Considering the regression in logarithmic scale, log (It) = (b0 + b1t), $APC=100\;x\;(e^{b}-1)$ 95% confidence interval was calculated using the parametric method.

The models were evaluated with and without autocorrelation (AC) term, and the term was maintained when the APC changed by more than 0.2%.

Time trends were estimated according to sociodemographic variables for the two indicators: the proportion of women who had seven or more prenatal care visits, from 2000 to 2020; and the proportion of women in the categories "did not/inadequate" and "adequate/more than adequate" access, from 2014 to 2020. It is worth highlighting that, since temporal behavior may differ between variables, and between the categories of the same variable, the resulting periods are not always the same.

In order to assess inequalities, measures of association (absolute and relative differences) were calculated for the two indicators (proportion of seven or more prenatal care visits; proportion of adequacy of access), according to the variables "age", "schooling" and "race/skin color", in 2014 and 2020. The differences were calculated using the categories that showed the best results - the highest proportion of the indicator - as a reference. To estimate the absolute difference (AD), the values of the proportions between the extreme categories (the highest and the lowest values of the indicator) were subtracted; and for the relative difference (RD), the ratio between the proportions of the same categories (the highest and lowest values). When the absolute and relative differences of the proportions of the extreme categories of the variables are close to zero and 1, respectively, they indicate absence of inequality.^27^

This study is part of the research *Study on indicators of women's and children's health in the health regions of the state of Rio de Janeiro*, approved by the Research Ethics Committee of the Universidade Federal Fluminense [Certificate of Submission for Ethical Appraisal (CAAE) No. 29721320.0.0000.5243], Opinion No. 4,091,556, issued on June 16, 2020.

## Results

In the period from 2000 to 2020, in Baixada Litorânea, there were 207,325 live births, of single fetus, of these 8,563 in 2000 and 10,808 in 2020. In that period, the information on the number of prenatal care visits marked with ignored had a percentage of less than 2%.

The proportion of women who had seven or more prenatal care visits increased from 51.7% in 2000 to 63.4% in 2020. There was stability between 2000 and 2013, and a significant upward trend between 2013 and 2020 (2.4% per year). When analyzed according to sociodemographic variables, the trend was differentiated in magnitude (APC) and direction (Table 1).

**Table 1:** Proportion of women with seven or more prenatal care visits and temporal trend, according to sociodemographic variables, Baixada Litorânea, state of Rio de Janeiro, Brazil, 2000-2020

Variable	2000 n = 8,563	2020 n = 10,808	Anual percentage change (95%CI^a^)	Trend
Total	51.7	63.4	2000-2003: 3.5 (-1.2;8.5)	Stability
			2003-2013: -0.2 (-1.1;0.6)	Stability
			2013-2020: 2.4 (1.1;3.7)	Increase
**Maternal age (in years)**				
≤ 19	45.1	51.4	2000-2004: 3.3 (-0.9;7.8)	Stability
			2004-2007: -7.4 (-19.0;5.7)	Stability
			2007-2020: 1.8 (1.1;2.5)	Increase
20-34	53.4	64.2	2000- 2004: 3.1 (0.4; 5.9)	Increase
			2004-2009: -1.2 (-3.9;1.5)	Stability
			2009-2020: 1.3 (0.7;1.9)	Increase
≥ 35	56.5	71.1	2000-2020: 1.0 (0.6;1.3)	Increase
**Schooling (in years of study)**				
No	35.0	25.0	2000-2020: -0.9 (-3.4;1.7)	Stability
1-3	34.9	37.2	2000-2020: -0.2 (-1.2;0.7)	Stability
4-7	46.5	47.8	2000-2013: -1.4 (-2.4;-0.5)	Decrease
			2013-2020: 2.7 (0.2;5.3)	Increase
8-11	57.5	62.6	2000-2003: 2.9 (0.1;6.0)	Increase
			2003-2009: -2.0 (-3.2;-0.7)	Decrease
			2009-2020: 1.3 (1.0;1.9)	Increase
≥ 12	80.8	81.9	2000-2004: -1.5 (-3.5;0.6)	Stability
			2004-2020: 0.5 (0.2;0.8)	Increase
**Race/skin color**				
White	57.5	70.0	2000-2020: 0.8 (1.2;6.1)	Increase
Mixed race	39.7	60.3	2000-2002: 12.2 (1.0;24.7)	Increase
			2002-2007: -2.5 (-5.7;0.9)	Stability
			2007-2020: 2.7 (2.1;3.3)	Increase
Black	44.5	61.1	2000-2020: 1.6 (1.0;2.2)	Increase

a) 95%CI: 95% confidence interval.

All age groups showed an upward trend at some point. For adolescents girls, this trend was significant only in the period from 2007 to 2020. Those aged ≥ 35 years old showed an upward trend throughout the period. The higher the maternal age, the higher the proportion of having seven or more prenatal care visits. Women with less than eight years of schooling showed a predominant pattern of stability. Those with eight or more years of schooling, on the other hand, had an upward trend. The higher the level of schooling, the higher the proportion of seven or more prenatal consultations. It could be seen an upward trend for all categories of race/skin color, being more intense for mixed race women between 2007 and 2020, and stable for White and Black women. White women maintained the highest percentage throughout the period (Table 1).

The category of adequacy of access "more than adequate" was predominant in the period, ranging from 45.4% (2014) to 52.8% (2020); we added to this, the category "adequate", in which 60.9% of women were included in 2020. The average percentage of records that were not classified was 12.2% in the period. When only full-term live births were analyzed, the numbers were similar (Table 2).

**Table 2:** Proportion of women according to adequacy of access to prenatal care, for the total number of live births (LB) and full-term LB, in Baixada Litorânea, state of Rio de Janeiro, Brazil, 2014-2020

Classification	2014	2015	2016	2017	2018	2019	2020
All live births^a^	n = 11,028	n = 11,465	n = 10,879	n = 11,186	n = 11,508	n = 11,276	n = 10,808
Did not	0.3	0.3	0.4	0.4	0.5	0.4	0.4
Inadequate	22.7	21.8	21.8	19.7	17.6	18.2	17.6
Intermediate	8.8	8.5	8.2	9.1	8.0	8.3	9.8
Adequate	8.7	9.0	8.4	8.8	7.8	7.5	8.1
More than adequate	45.4	46.3	48.3	50.7	54.1	56.0	52.8
Not classified	14.1	14.1	12.9	11.3	12.0	9.6	11.3
Full-term live birth^b^	n = 9,819	n = 10,304	n = 9,654	n = 10,016	n = 10,329	n = 10,039	n = 9,647
Did not	0.3	0.3	0.3	0.4	0.4	0.4	0.3
Inadequate	22.1	21.4	21.3	19.5	17.5	18.1	17.3
Intermediate	7.9	7.5	7.2	8.3	6.9	7.3	8.8
Adequate	8.4	8.6	8.0	8.4	7.4	7.1	7.9
More than adequate	47.0	47.4	50.0	52.1	55.7	57.5	54.3
Not classified	14.3	14.8	13.2	11.4	12.1	9.6	11.4

a) LB: Single fetus, weight ≥ 500 g and gestational age ≥ 22 weeks; b) Full-term LB: Single fetus, weight ≥ 500 g and gestational age ≥ 37 weeks.

In the time series between 2014 and 2020 (Table 3), there was a prevalence of an increasing trend in the categories "adequate" and "more than adequate", and a decrease in inadequacy. Although the favorable changes, about 25% of adolescent girls remained in the category "did not/inadequate" and just over 50% achieved adequacy. Women aged 35 years and older, despite their stability, always showed more than 60% adequacy. The 20 to 34 age group had intermediate result, that is, the adequacy of access gradually increased according to the mother’s age. Similarly, the higher the number of years of schooling, the higher the proportion of women with adequate/more than adequate prenatal care. Women of all race/skin colors showed an upward trend in adequacy, however, White women stood out showing as they presented the highest percentage of adequate/more than adequate (always higher than 60%) (Table 3).

**Table 3:** Proportion of women by category of adequacy of access to prenatal care (did not/inadequate versus adequate/more than adequate,^a^ and temporal trend, total and according to sociodemographic variables, Baixada Litorânea, state of Rio de Janeiro, Brazil, 2014-2020

Classification/variables	2014	2020	Annual percetage change (95%CI^a^)	Trend
**Total of pregnant women**				
Did not/inadequate	22.4	17.1	2000-2020: -4.3 (-5.9;-2.7)	Decrease
Adequate/more than adequate	55.4	62.2	2000-2020: 2.6 (1.2;4.0)	Increase
**Age (in years)**				
**10-19**				
Did not/inadequate	30.6	25.1	2000-2020: -3.1 (-4.8;-0.1)	Decrease
Adequate/more than adequate	44.0	52.6	2000-2020: 3.7 (2.0;5.3)	Increase
**20-34**				
Did not/inadequate	21.2	16.8	2000-2020: -4.1 (-5.9;-2.2)	Decrease
Adequate/more than adequate	57.4	63.4	2000-2020: 2.2 (1.0;3.5)	Increase
**≥ 35**				
Did not/inadequate	15.8	14.3	2000-2020: -3.1 (-6.0;0.0)	Stability
Adequate/more than adequate	62.3	65.6	2000-2020: 1.9 (-0.3;4.2)	Stability
**Schooling (in years of study)**				
**≤ 7**				
Did not/inadequate	32.3	30.4	2000-2020: -0.3 (-3.5;3.1)	Stability
Adequate/more than adequate	41.1	47.2	2000-2020: 2.8 (0.4;5.3)	Increase
**8-11**				
Did not/inadequate	21.0	16.4	2000-2020: -4.1 (-5.9;-2.2)	Decrease
Adequate/more than adequate	56.4	62.8	2000-2020: 2.2 (0.8;3.7)	Increase
**≥ 12**				
Did not/inadequate	11.6	8.8	2014-2018: -9.5 (-17.0;-1.4)	Decrease
			2018-2020: 5.2 (-21.1;40.2)	Stability
Adequate/more than adequate	72.7	75.0	2000-2020: 0.6 (-0.1;1.3)	Stability
**Race/skin color**				
**White**				
Did not have prenatal care visits/inadequate	18.9	12.3	2000-2020: -6.1 (-9.8;-3.1)	Decrease
Adequate/more than adequate	61.3	67.6	2000-2020: 1.9 (0.3;3.5)	Increase
**Black**				
Did not have prenatal care visits/inadequate	28.2	19.2	2000-2020: -5.6 (-8.7;-2.5)	Decrease
Adequate/more than adequate	47.6	61.1	2000-2020: 4.1 (3.1;5.2)	Increase
**Mixed race**				
Did not have prenatal care visits/inadequate	25.2	19.8	2000-2020: -4.1 (-5.4;-2.8)	Decrease
Adequate/more than adequate	49.6	60.3	2000-2020: 3.8 (2.0;5.5)	Increase

a) The intermediate category was not included.

Between 2014 and 2020, the absolute differences in having seven or more prenatal consultations decreased between women ≥ 35 years old and other age groups. It is worth mentioning that absolute inequality for adolescents was still high, however, the relative difference decreased. Compared to the group ≥ 12 years of schooling, the absolute difference decreased in women of other levels of schooling; with the exception of the group between 0 and 3 years, which maintained both inequalities. Absolute and relative differences between White, mixed race and Black decreased (Table 4).

**Table 4:** Absolute and relative differences in having seven or more consultations and the adequacy of access to prenatal care, according to sociodemographic variables, Baixada Litorânea, state of Rio de Janeiro, Brazil, 2014-2020

Variable	Proportion of 7 or more prenatal consultations						Proportion of adequacy of access to prenatal care (full-term LB)					
	2014 (n = 11,028)			2020 (n = 10,808)			2014 (n = 10,913)			2020 (n = 9,647)		
		Differences			Differences			Differences			Differences	
	%	Absolute	Relative	%	Absolute	Relative	%	Absolute	Relative	%	Absolute	Relative
**Age (in years)**															
≤ 19	43.3	24.1	1.6	51.4	19.7	1.4	42.1	19.4	1.5	52.6	13.0	1.2
20-34	60.1	7.2	1.1	64.2	6.9	1.1	56.2	5.3	1.1	63.4	2.2	1.1
≥ 35^a^	67.4	-	-	71.1	-	-	61.5	-	-	65.6	-	-
**Schooling (in years of study)**															
0-3	34.9	45.9	2.3	35.8	46.1	2.3	33.3	39.5	2.2	40.4	34.6	1.9
4-7	41.7	39.1	1.9	47.8	34.1	1.7	42.2	30.5	1.7	47.7	27.3	1.6
8-11	61.2	19.6	1.3	62.6	19.3	1.3	56.4	16.4	1.3	62.8	12.2	1.2
≥ 12^b^	80.8	-	-	81.9	-	-	72.7	-	-	75.0	-	-
**Race/skin color**															
Mixed race	52.8	13.2	1.3	60.3	9.7	1.2	49.6	11.7	1.2	60.3	7.3	1.1
Black	52.1	12.5	1.2	61.1	8.9	1.1	47.6	13.7	1.3	61.1	6.5	1.1
White^c^	65.3	-	-	70.0	-	-	61.3	-	-	67.6	-	-

a) Reference category of maternal age for the calculation of differences; b) Reference category of maternal schooling for the calculation of differences; c) Reference category of maternal race/skin color for the calculation of differences.

Absolute and relative inequalities in the adequacy of access to prenatal care decreased for all age groups, levels of schooling and race/skin color. (Table 4); however, they remained high among women with low level of schooling and adolescents.

**Figure 1:**
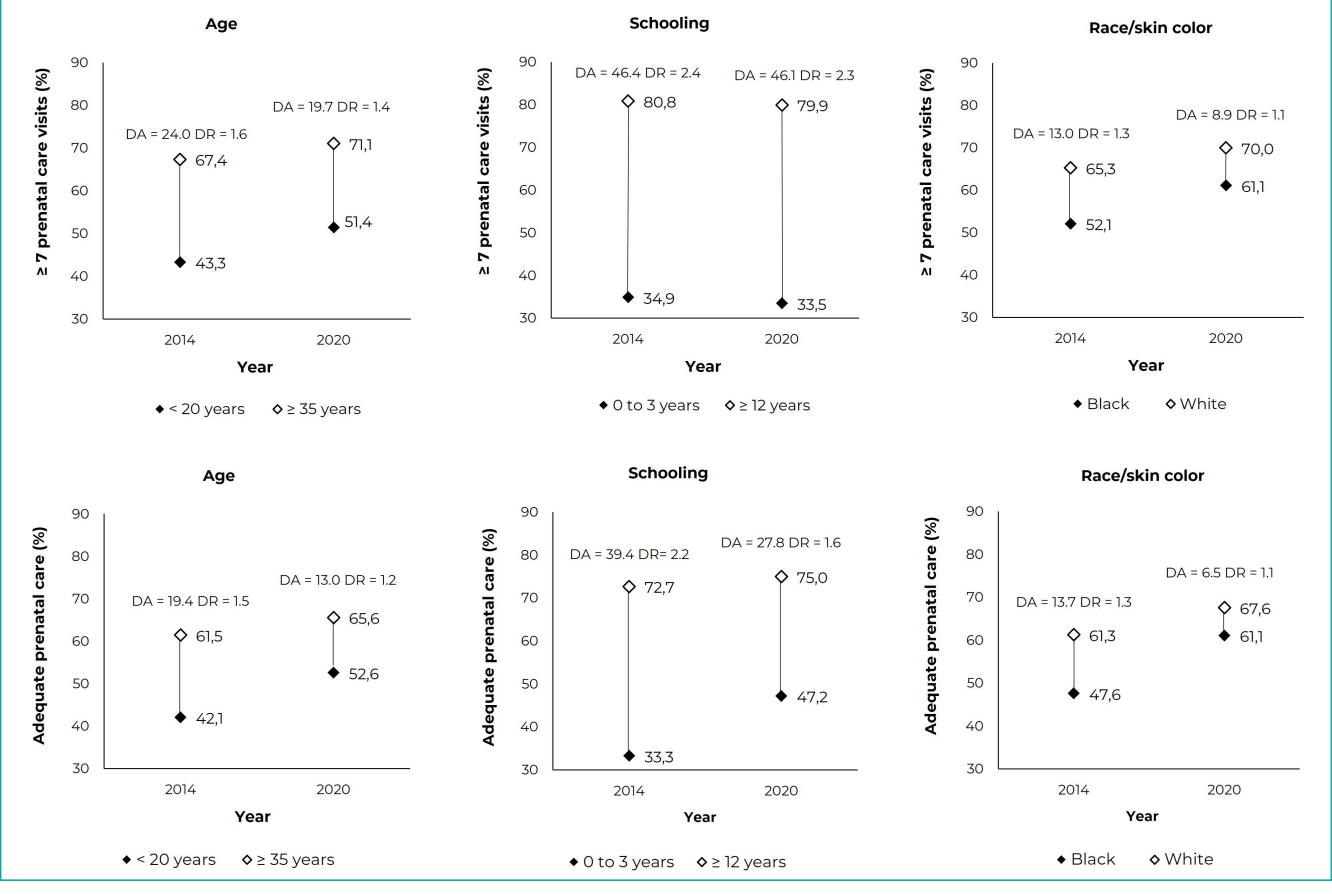
Proportions of seven or more prenatal consultations (upper graphs) and adequacy of adequate/more than adequate access to prenatal care (lower graphs) and their absolute differences (ADs) and relative differences (RDs), according to extreme categories of sociodemographic variables, Baixada Litorânea, state of Rio de Janeiro, Brazil, 2014-2020

The absolute and relative differences in indicators - proportion of ≥ 7 prenatal care visits (2014 and 2020) and proportion of adequate prenatal care (2014 and 2020) - by extreme categories of maternal age, schooling and race/skin color, are expressed in Figure 1. Despite the upward trend of indicators in all categories, a higher magnitude was observed in mothers aged 35 years and older, with 12 or more years of schooling and of White race/skin color, when compared to those who were less favored. The greatest absolute differences were observed for schooling, either regarding the number of prenatal consultations or in the adequacy of access.

## Discussion

This study identified an increase in both the number of visits and the adequacy of access to prenatal care in Baixada Litorânea, state of Rio de Janeiro. However, this increase proved to be insufficient in view of the established target aimed at 70% for the first indicator,^14^ and the persistence of inequalities in both indicators. Adolescents, women with less than eight years of schooling and those of Black race/skin color showed the most unfavorable results, whose differences were attenuated but not reversed during the study period.

There was an increasing trend in the category of seven or more prenatal care visits, until reaching 63.4%, in 2020. For Brazil, in 2013, this indicator was 63.2% for single pregnancies, although in the Southeast region it had reached 73%, almost ten percentage points higher than that found for Baixada Litorânea;^13^ data on Brazil, in 2015, showed a 66.9% increase.^13^ When comparing Baixada Litorânea to other locations, only Fortaleza, state of Ceará, showed lower percentages, 56.8%, while Campinas and São Paulo, state of São Paulo, and Porto Alegre, state of Rio Grande do Sul, had between 74% and 79% in 2015-2016.^11^ The analysis of the temporal trend in Baixada Litorânea only identified an increase in the period from 2013 to 2020, probably resulting from measures of the Maternal-Child Health Services strategy, implemented in 2011 at the national level.^5^ However, it is necessary to maintain or increase this growth rate in order to reach, at least, the level of the Southeast region in the coming years.

The indicator of seven or more prenatal care visits had an upward gradient as maternal age increased; however, adolescent girls comprised a disadvantaged group, with the lowest percentage, while older women had the highest values, a behavior similar to that showed by a 2013 national study.^13^ It is noteworthy that in Baixada Litorânea fluminense, the values for adolescents were lower than those at the national level.^13^ Women with less than eight years of schooling showed the lowest proportions, always less than 50%, while those with ≥ 12 years of schooling reached the highest value, 81.9% in 2020, a finding similar to those of national studies.^9^ ^,^ ^10^ In Baixada Litorânea, however, there was stagnation in the indicator, contrary to what occurred in Brazil, between 2000 and 2015, when there was a more significant increase in the low level of schooling groups, about 3.2% per year.^10^

The percentage of having seven or more prenatal consultations among White women was always higher than that of mixed race and Black women, as at the national level.^9^ ^,^ ^10^ However, White women in Baixada Litorânea only reached 70%, a proportion lower than national values;^9^ ^,^ ^10^ The fact that Baixada Litorânea did not accompany improvements in the use of prenatal care suggests failures in the recruitment and follow-up of pregnant women, especially the most vulnerable ones. In turn, regional characteristics regarding the configuration of vulnerabilities could explain, although partially, this scenario that may support managers in promoting maternal and child health actions. For example, adolescents who get pregnant have been characterized as a more vulnerable group, including other factors such as low level of schooling and low income, which also potentiate lower access to and use of prenatal and perinatal care.^23^

There are still few studies using the indicator of adequacy of access to prenatal care.^11^ ^,^ ^13^ The values reported for the sum of the categories "adequate" and "more than adequate", at the national level, related to 2015, were higher in the South and Southeast regions (> 76%), and lower in the Northeast (63.8%) and North (53.1%) regions. The Midwest region (71.7%) showed a value closer to that found for Brazil (70.2%).^13^ The state of Rio de Janeiro, where the studied region is located, showed the lowest value in the Southeast region, 70%, although Baixada Litorânea ranged from 55% to 62%, a pattern similar to that of the North and Northeast regions of the country. It should be noted that Baixada Litorânea performed better in the item "beginning of prenatal care", given that about 80% of the women in the region have been able to start prenatal care during the first trimester (data not shown in the table), however there is a lack of improvement of the longitudinality of this care.

When compared to data of the categories "adequate" and "more than adequate" related to Brazil in the years 2014 and 2015,^13^ the pattern in Baixada Litorânea was more unfavorable for adolescents, Black and mixed race women, and those with low level of schooling, all of them showing values lower than the national ones,^13^ presenting greater vulnerability for this region of the state of Rio de Janeiro. Regional characteristics regarding the organization of services, implementation of the Maternal-Child Health Services and distribution of vulnerabilities among the population may explain why this region remains with indicators lower than those of the rest of the state.^14^

As an alternative to assess inequality in prenatal care, a national study explored the relative and absolute differences in the period from 2000 to 2015.^10^ We identified a decrease in both types of differences for having seven or more prenatal consultations, in relation to schooling and race/skin color, without analyzing maternal age. According to the present study in Baixada Litorânea, adopting the same approach, although for a shorter period, from 2014 to 2020, inequalities had been attenuated, however persisted: in schooling, the behavior of bottom inequality,^27^ or marginal exclusion was observed, given that women with 12 or more years of schooling showed adequate coverage, and there are great relative differences, that is, the lower the level of schooling, the greater the differences. The differences were of smaller magnitude when comparing race/skin color, and decreased for mixed race and Black women.

Finally, inequality, both relative and absolute, decreased for adolescents, however, still remained high, corroborating the vulnerability of this age group. It is worth noting that these patterns of inequalities have been identified in other studies on women's health, showing that there is a harmful accumulation in the reproductive period.^15^ ^,^ ^21^ ^,^ ^22^

The limitations of this study are related to the quality of data obtained from information systems. An analysis of SINASC, at the national level (2019), showed a slight overestimation of the percentage of seven or more prenatal visits and high level of schooling (12 and more years of schooling), with good agreement: Kappa coefficient of 0.639 and 0.680, respectively.^28^ Another limitation of this work is associated to the indicator, only quantitative, given that it is not possible to explore in depth the quality of care based only on the number of consultations. However, the results of recent national studies, with more refined indicators of adequacy of prenatal care, identified the same inequalities: association between inadequate prenatal care and less than 20 years of age, less than eight years of schooling and Black race/skin color.^6^ ^,^ ^15^ ^-^ ^18^ Finally, by including the year 2020, there may have been changes in the trend analysis, since all health services suffered the impact of the COVID-19 pandemic, including prenatal care.^29^ After all, we analyzed the series without and with the year 2020, and the differences were small, although it is worth highlighting a decrease in the number of prenatal visits in 2020.

As a strength of this study, it was the first to analyze the use and adequacy of access to prenatal care in Baixada Litorânea, state of Rio de Janeiro. The regional analysis is aligned with the way the management of the Maternal-Child Health Services is organized and may support the necessary changes. The power of the SINASC was confirmed as a data source for studies in the maternal and child area, as well as for monitoring health indicators and inequalities. The use of absolute and relative differences strengthened the analysis of inequalities and can be incorporated into further studies.

It can be concluded that sociodemographic inequalities in prenatal care persist. In the trend assessment, inverse equity was observed, that is, first the new interventions reach the most favored groups and, then, the less favored, with persistence of inequalities.^30^ Baixada Litorânea, a region of the state of Rio de Janeiro, is more disadvantaged than other places where inequalities have been reduced,^17^ ^,^ ^20^ pointing to the need to prioritize care for pregnant women with low level of schooling, those of Black or mixed race/skin color andadolescents.
